# Simple Preparation of Metal-Impregnated FDM 3D-Printed Structures

**DOI:** 10.3390/mi13101675

**Published:** 2022-10-04

**Authors:** Diana Flores, Jose Noboa, Mickaela Tarapues, Karla Vizuete, Alexis Debut, Lorena Bejarano, Daniela Almeida Streitwieser, Sebastian Ponce

**Affiliations:** 1 Department of Chemical Engineering, Universidad San Francisco de Quito USFQ, Diego de Robles s/n y Avenida Interoceánica, Quito 170157, Ecuador; 2 Department of Materials Engineering Science, Graduate School of Engineering Science, Osaka University, Toyonaka, Osaka 560-8531, Japan; 3 Centro de Nanociencia y Nanotecnología, Universidad de las Fuerzas Armadas ESPE, Sangolquí 171103, Ecuador; 4 Department of Mechanical Engineering, Universidad San Francisco de Quito USFQ, Diego de Robles s/n y AvenidaInteroceánica, Quito 170157, Ecuador; 5 Faculty for Applied Chemistry, Reutlingen University, 72762 Reutlingen, Germany

**Keywords:** 3D-printing, FDM, silver nanoparticles, metal impregnation

## Abstract

Modifying the natural characteristics of PLA 3D-printed models is of interest in various research areas in which 3D-printing is applied. Thus, in this study, we describe the simple impregnation of FDM 3D-printed PLA samples with well-defined silver nanoparticles and an iron metal salt. Quasi-spherical and dodecahedra silver particles were strongly attached at the channels of 3D-printed milli-fluidic reactors to demonstrate their attachment and interaction with the flow, as an example. Furthermore, Fenton-like reactions were successfully developed by an iron catalyst impregnated in 3D-printed stirrer caps to induce the degradation of a dye and showed excellent reproducibility.

## 1. Introduction

Interest in the additive manufacturing process known as 3D printing has grown in different scientific areas in recent years [1,2], due to the advantages of rapid prototyping, fast design, ease of access, cost effectiveness, among many others. This fast development process has been applied in batch and micro- and milli-fluidic reactors [3,4,5,6] for chemical synthesis, medical devices [7], medicine tablets for drug delivery [8,9], 3D-printed scaffolds for tissue engineering [10], laboratory equipment [11], analytical and bioanalytical sensors [12], catalytic systems [13,14], etc. Among several 3D-printing techniques, fused deposition modeling (FDM) appears to be the cheapest, fastest, and most affordable method [1]. FDM uses a variety of thermoplastics, such as polycarbonate, acrylonitrile butadiene styrene, glycol modified polyethylene terephthalate, etc. Polylactic acid (PLA) is a thermoplastic produced from renewable resources and probably the most often used polymer for 3D-printing. As a thermoplastic, it is chemically inert, which can be beneficial or limiting depending on its application. To address limitations, several approaches have been presented for modifying the natural characteristics of PLA 3D-printed models. The most common techniques are mechanically mixed dyes, soluble drugs, and metal salt precursors mixed with the polymer before hot melting extrusion [8,15]. Additionally, some approaches to the surface functionalization of 3D-printed scaffolds with NaOH + EDC/NHS for impregnating bioactive molecules were also developed [10].

Interest in the introduction of metal particles at the PLA surface for imprinting antimicrobial, antibacterial, optical, and sensing properties has increased in recent years. The use of silver nanoparticles (AgNPs) has (arguably) been the most representative, due to their exceptional optical, electrical, antimicrobial, antifungal, and antiviral properties [16,17]. Furthermore, the use of 3D-printed catalytically active devices for heterogeneous reactions in synthetic chemistry was also demonstrated, but for more complex and costly 3D-printed methods [13]; these are not affordable for most laboratories [18]. Therefore, research focused on the impregnation of FDM-printed structures with well-defined ex-situ synthesized particles or a variety of metals for different applications is still missing.

**Figure 1:**
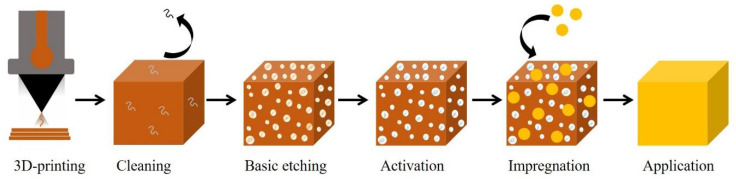
Printing, etching, and impregnation of 3D-printed samples developed in this work.

In this work, the possibility of impregnating simple metal catalysts, as well as highly defined metal nanoparticles under PLA surfaces, following the method shown in Figure 1, was explored. PLA samples such as milli-reactors and stirrer caps were obtained using a low-cost FDM 3D-printer. The attachment, particle interaction, and catalytic activity of 3D-printed devices was demonstrated.

## 2. Materials and Methods

### 2.1. Materials

Silver nitrate (AgNO_3_) (reagent grade, Scharlau), sodium borohydride (NaBH_4_) (98%, Acros Organics), sodium citrate (Na_3_C_6_H_5_O_7_) (reagent grade), hydrogen peroxide (H_2_O_2_) (30%, Fischer Chemical), and distilled water were used for the synthesis of the silver nanoparticles.

Potassium hydroxide (KOH) and sodium carbonate (Na_2_CO_3_) were used for the etching process of the polymer surfaces. Iron (III) Chloride Hexahydrate (FeCl_3_ 6H_2_O) was used for catalyst impregnation on the polymer surfaces. Methylene blue was used for the decolorization experiments. All reagents were employed as received without further purification.

### 2.2. Ex-Situ Synthesis of Ag Colloids

The synthesis procedure of quasi-spherical and variable shape AgNPs was adjusted from a previous contribution [3]. Freshly prepared AgNO_3_ (10 mM), NaBH_4_ (100 mM), Na_3_C_6_H_5_O_7_ (100 mM), and H_2_O_2_ (400 mM) were used as starting solutions. In an Erlenmeyer flask containing 250 mL of distilled water, a defined volume of each solution was added. First, 2.5 mL of trisodium citrate (100 mM) and 2.5 mL of NaBH_4_ (100 mM) were mixed. Then, 3.0 mL of aqueous AgNO_3_ (100 mM) was added. The solution was vigorously stirred for 20 min. A yellow colloid was obtained for the quasi-spherical particles. This colloid was used for impregnation, but also as the starting solution for non-spherical particles synthesis.

Bigger clusters were synthesized using a 3D-printed reactor illuminated with a green LED light (100 W, IP66) at room temperature [3]. Briefly, the starting solution was pumped through the reactor ware with an infusion syringe pump (SP-200, Advanced Instrumentation, Miami, FL, USA) for 4 h. Samples were collected and stored in the dark prior to characterization.

### 2.3. Etching and Impregnation of 3D-Printed Samples

Figure 1 shows the general procedure of etching and impregnation of the 3D-printed samples. The 3D samples were printed in polylactic acid (PLA) using an affordable 3D printer (WEEDO 152 s), which deposited layers (layer height = 0.2 mm) of thermopolymers via an extruder nozzle.

The polymer surface etching process was based on the procedure developed by Bernasconi et al. [19]. Briefly, the procedure was as follows: i. The samples were cleaned in a 50 g L^−1^ (Na_2_CO_3_) solution for 10 min, and then washed with distilled water; ii. the cleaned samples were embedded in 40 g L^−1^ of KOH for 75 min, and then washed with distilled water; iii. the samples were activated in a 20 g L^−1^ of NaBH_4_ for 2 min, and then washed with distilled water; iv. finally, the samples were impregnated. For impregnation of the NPs, colloids were titrated with a 2 mM solution of NaOH to pH > 10 (time: 48 h).

For iron impregnation, a 0.75 M solution of FeCl_3_ 6H_2_O was prepared. For both impregnation processes, the samples were embedded in the catalyst solution with continuous stirring for at least three days to obtain a homogeneous surface. Iron-impregnation was developed without headspace to prevent Fe^2+^ oxidation and precipitation [20]. For iron impregnated structures, just after wet impregnation, the samples were dried at 60 °C for 4 h in a convective oven.

### 2.4. Dye Degradation

A 0.125 mM MB and 0.01 mM H_2_O_2_ aqueous solution was prepared. Then, the non-and impregnated 3D-printed stirrer caps were placed. Samples were taken every 2 min and analyzed by UV/Vis spectroscopy.

### 2.5. Analytical Methods

The absorption spectra of Ag colloids and selected decolorization experiments were recorded using a UV/Vis spectrometer (CE 204, CECIL, Buck Scientific, Norwalk, CT, USA) from 300 to 1000 nm.

For the study of the size and morphology of the silver nanoparticles (AgNPs), a FEI Spirit Twin with LaB6 filament transmission electron microscope (TEM) was used operating at a voltage of 80 kV.

Materials surface analysis was developed in a Tescan Mira 3 microscope equipped with a Schottky Field Emission Gun (Schottky FEG-SEM) that allows us to obtain a resolution of 1.2 nm at 30 keV. The elemental analysis was obtained by Energy Dispersive Spectroscopy (EDS), which was performed on the SEM chamber at 30 kV using a Bruker X-Flash 6|30 detector, with a 123 eV resolution at Mn Kα.

The crystallographic structure was determined by X-Ray Diffractometry (XRD). The XRD was carried out using an Empyrean diffractometer from PANalytical operating in a θ–2θ configuration (Bragg-Brentano geometry) and equipped with a Cu X-ray tube (Kα radiation λ = 1.54056 Å) operating at 40 kV and 40 mV.

## 3. Results and Discussion

**Figure 2:**
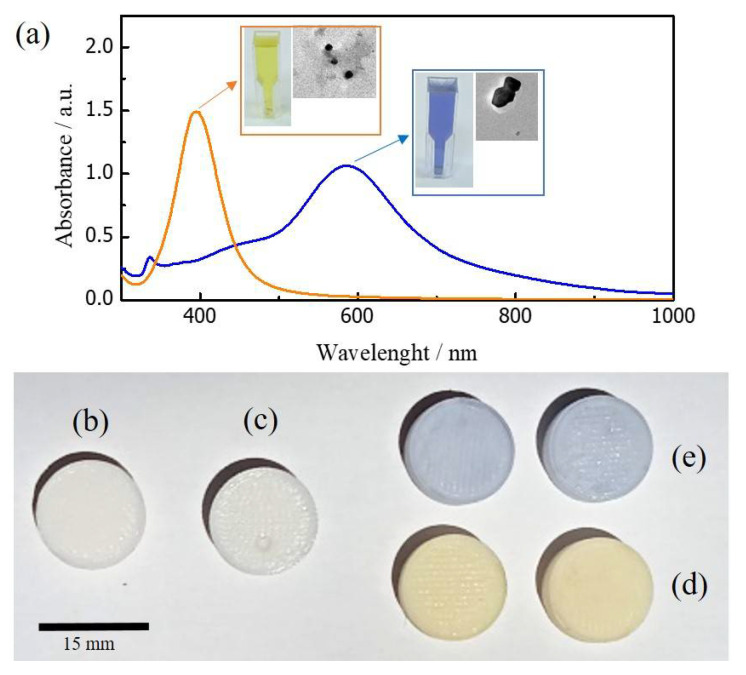
Top: (**a**) UV/Vis spectra of ex-situ synthesized catalyst. Orange spectrum: standard AgNPs, blue spectrum: photo-illuminated bigger clusters. Insets: Colloid picture and exemplary TEM image of AgNPs. Bottom: (**b**) 3D-printed sample, (**c**) 3D-printed sample without etching, but through impregnation procedure. 3D-samples after etching and impregnation process with standard (**d**) and photosynthesized Ag clusters (**e**).

The surface plasmon resonance (SPR) spectra of both types of ex-situ synthesized silver nanoparticles is shown in Figure 2a. Standard AgNPs (orange line spectrum) indicate a SPR maxima around 400 nm, which is related to quasi-spherical NPs (average particle size ≈ 15 nm). In contrast, photosynthesized clusters (blue line spectrum) show an SPR maximum at 585 nm, which is representative of the presence of decahedral structures (average particle size > 20 nm). Please refer to our previous contribution [3] for a complete nanoparticles synthesis procedure and analysis.

For comparison, 3D-printed samples without an etching process were exposed to the silver colloids for a long period of time (around 48 h). These samples did not adsorb the clusters, keeping the same surface characteristics as 3D-printed-only structures (Please refer to Figure 2b,c). In contrast, 3D-printed samples with an etching process adsorbed both silver clusters at the PLA surface during the impregnation process. Both samples show a colored appearance depending on the colloid impregnation, as shown in Figure 2d,e.

**Figure 3:**
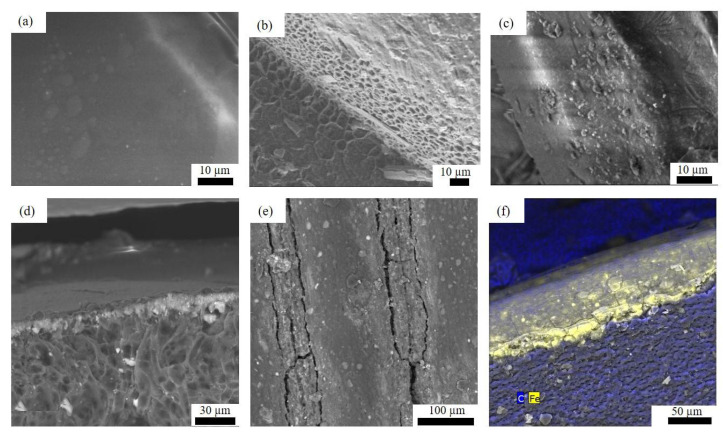
SEM images of a PLA 3D-printed surface (**a**) before, (**b**) after etching process, (**c**) etching process and NPs impregnation, (**d**) cross-section of NPs impregnated surface, (**e**) etching process and Fe impregnation, and (**f**) cross-section of an Fe-impregnated surface.

For a better understanding of the etching and impregnation process, SEM images of the 3D-printed samples before and after the procedure were taken (Figure 3). The PLA 3D-printed surface shows a smooth texture without the presence of irregularities at the macroscale. Nevertheless, after the etching process, the smooth PLA surface (Figure 3a) is transformed into a porous structure (Figure 3b), due to the strong alkali attack.

Interestingly, as depicted in Figure 2c, the AgNPs appear to occupy the new PLA porous surface during the impregnation procedure developing a thin NPs layer at the PLA surface, as shown in the cross-section image in Figure 3d. AgNPs were not detached after cleaning the samples with water. For further confirmation, the change in surface elemental composition was analyzed by EDS before and after the impregnation process, and the results are shown in Table 1. The peak element of Ag clearly appears, reducing the amount of C and O detected at the impregnated surface.

**Figure 4:**
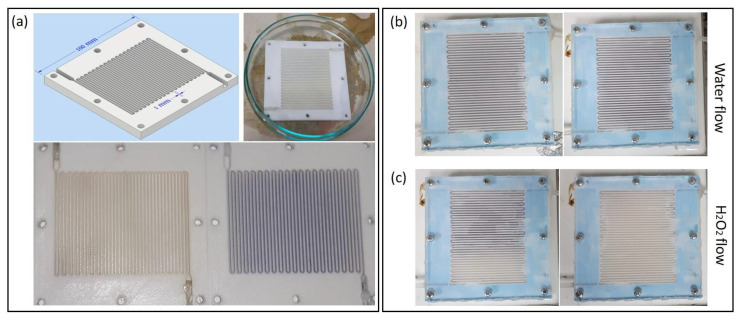
(**a**) Top-left: CAD design of 3D-printed milli-reactor, Top-right: impregnation process of 3D-printed milli-reactor, Bottom: 3D-printed milli-reactors impregnated with spherical AgNPs (yellow hue) and non-spherical AgNPs (blue hue), respectively. (**b**) Image of impregnated 3D-printed PLA milli-reactors before (**left**) and after (**right**) long-time water stream. (**c**) Image of impregnated 3D-printed PLA milli-reactors before (**left**) and after (**right**) hydrogen peroxide stream.

For testing the AgNPs attachment to the PLA surface, 3D-printed milli-reactors (1 mm channel width) were impregnated in the channels with both synthesized colloids, (Figure 4a) (Please refer to a previous contribution [3] for a complete description of the design and assembly of the 3D-printed milli-reactors and pumping system). In our previous contribution, the 3D-printed reactors did not adsorb NPs when they were pumped across the fluidic system. Thus, for impregnation, the procedure specified above was required. Once the impregnated reactors were prepared, a water stream was continuously pumped (2 mL h^−1^) overnight. No NPs detachment was observed following the water stream procedure (see Figure 4b), showing the strong adhesion of clusters at the PLA surface during continuous flow. Moreover, it is well-known that AgNPs strongly interact with hydrogen peroxide in a Fenton-like reaction generating hydroxyl radicals and Ag^+^ [21]. As a result, NPs are reduced and dissolved in the medium. This phenomenon was tested by pumping an H_2_O_2_ water solution (0.2 mM) along the impregnated reactor. As shown in Figure 4c, NPs strongly interacted with H_2_O_2_, as clusters disappeared from the reactor channels during the flow. Both experiments showed the strong NPs attachment, but also their interaction with the flow, opening up different applications for simple PLA-coated surfaces, such as chemical and/or optical sensors, antimicrobial scaffolds, wall-coated microreactors for chemical synthesis, among many others.

**Figure 5:**
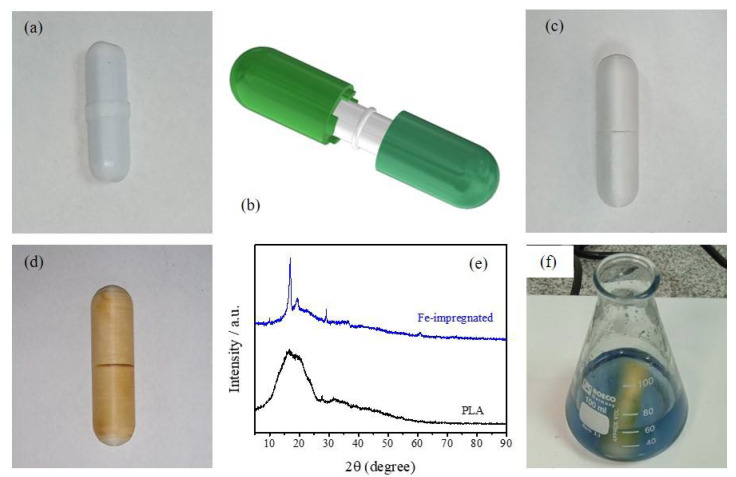
(**a**) Common lab stirrer, (**b**,**c**) schematic CAD representation and picture of a 3D-printed stirrer cap assembly, respectively, (**d**) Fe-impregnated stirrer cap assembly, (**e**) XRD pattern of PLA and Fe-impregnated surface, and (**f**) Fenton reaction with impregnated stirrer cap.

PLA-etched surfaces were also studied as devices for developing catalytic experiments. The possibility was explored of converting a stirrer to be catalytically active by the wet impregnation of an iron chloride salt. The device would present an advantage for developing heterogenous catalytic reactions without the need to remove or add a catalyst. For this purpose, a surrounding device was designed in CAD software, 3D-printed, and assembled over a common laboratory stirrer (Figure 5a–c). Once the 3D-printed stirrer cap assembly was obtained, caps were successfully wet-impregnated with Fe. Please refer to Figure 3e for a typical SEM image of PLA surfaces etched and impregnated with iron. Similar to the description above for metal nanoparticles, the porous surface was successfully covered by iron showing a yellowish appearance (Figure 5d), also developing a thin Fe layer over the PLA surface (see Figure 3f). As before, the presence of iron was successfully detected by EDS characterization (see Table 1). Moreover, Figure 5e shows the XRD patterns of PLA and Fe-impregnated surfaces. PLA showed the common broad spectra at 2θ = 16.5° for the semi-crystalline structure of it [22], while the crystalline components, due to Fe impregnation, are shown with the appearance of sharp peaks.

**Figure 6:**
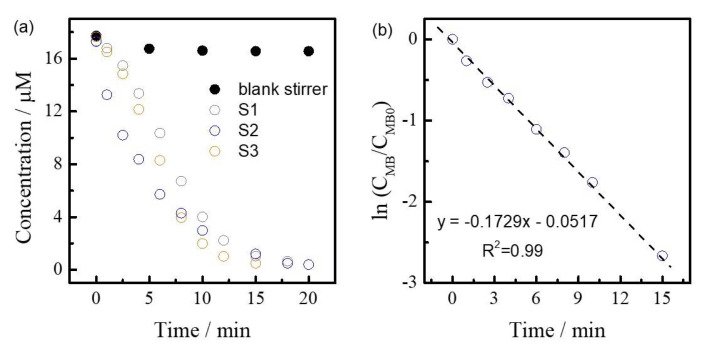
(**a**) Concentration vs. time curves of MB degradation with a non-impregnated and Fe-impregnated stirrer caps, and (**b**) fitting of MB degradation to a first order reaction kinetic model.

The Fe-impregnated 3D-printed stirrer caps were used for the degradation of methylene blue (MB) with hydrogen peroxide via an advance oxidation process in batch conditions (Figure 5f). The Fenton reaction has been widely reported in the literature [23], whereby the formation of hydroxide (OH^−^) and a hydroxyl radical by a reaction between Iron (II) (Fe^2+^) and hydrogen peroxide (H_2_O_2_) oxidizes contaminants. Concentration vs. time curves are depicted in Figure 6a for three different impregnated 3D-printed stirrer caps. For comparison, non-impregnated stirrer caps (black dots) did not show any catalytic activity. In contrast, Fe-impregnated stirrer caps were able to degrade MB obtaining a clear solution under 20 min, showing the expected catalytic activity of the system. Moreover, in accordance to Fenton-like reactions [24], MB degradation followed a pseudo-first order reaction mechanism (see kinetic fitting in Figure 6b). Notably, all impregnated stirrer caps exhibited similar calculated kinetic constants (*k* = 0.16 ± 0.01 min^−1^), demonstrating the reproducibility of the impregnation method. Furthermore, reusability tests were developed under the stirrers for testing metal impregnation at the PLA-surface. After one reaction, 3D-printed stirrer caps were cleaned and dried at 60 °C, and then the reaction was developed again. Notably, similar kinetic constants were calculated (±0.05 min^−1^), showing how good metals are attached to the PLA surface. This method opens up a simple and fast testing method for developing a variety of chemical syntheses at lab-scale.

## 4. Conclusions

This communication demonstrated the simple impregnation of 3D-printed PLA samples with well-defined ex-situ synthesized silver nanoparticles and an iron metal salt. However, the process should not be limited to them. Nanoparticles and iron showed to occupy the porous area generated after the etching process of PLA surfaces. Metal NPs showed a strong attachment to the PLA 3D-printed surfaces (i.e., channels in flow milli-reactors), but also interaction with H_2_O_2_ in continuous flow. Furthermore, the 3D-printed stirrer caps were successfully used as catalytically active devices for the degradation of a dye via an advanced oxidation process with excellent reproducibility.

All considered, we strongly believeF that this simple and affordable method for impregnating well-defined metals could open up applications in different scientific areas where 3D-printing is actually applied. In a future contribution, the method could be applied for performing more relevant catalytic conversions on 3D-printed small-scale reactors.

## Figures and Tables

**Table 1:** Elemental analysis (%) of PLA and impregnated samples.

Sample	Elemental Analysis (%)				
	C	O	Ag	Fe	* Others
PLA	52.87	47.13	-	-	
AgNPs-impregnated	42.03	20.71	33.98	-	3.29
Fe-impregnated	40.43	47.23	-	10.05	3.28

* Alkali metals present due to etching and impregnation processes.
